# Operation for the Ascites of Cirrhosis

**Published:** 1899-06-10

**Authors:** 


### OPERATION FOR THE ASCITES OF CIRRHOSIS.

About three years ago Mr. Rutherford Morison, of Newcastle-on-Tyne, described an operation which he had performed with the object of curing the ascites caused by cirrhosis of the liver. He had then performed the operation twice; now[1] he reports two more cases. To go back to the original report, the object was to relieve the obstruction of the portal circulation which occurs in connection with cirrhosis of the liver, an obstruction which is no doubt the cause of the effusion of fluid into the cavity of the peritoneum which, under the name of ascites, forms so important a factor in the downward progress of so many cases of the disease. Post-mortem examinations on a series of cases of hepatic cirrhosis had shown Dr. Drummond that in many cases in which ascites had been absent there existed vascular adhesions connecting the parietes to the viscera. In one such case the cirrhosis was known to have existed for nearly twenty years, and death eventually resulted from causes unconnected with it. In many of these cases a direct and ample communication between the portal and systemic veins was found, and this seemed to be a satisfactory explanation of the absence of ascites. Hence it was thought that if, in addition to the ordinary means of communication between the portal and the systemic circulations, wide adhesions could be produced between the viscera and the parietes which would become filled with vessels, a much more free anastomosis between the two circulations could be set up, and that in this way the occurrence of any material obstruction to the return of blood from the portal system might be prevented. With this object in view the abdomen was opened and dried out, the parietal peritoneum and that covering the opposed viscera, especially the liver and spleen, being scrubbed with sponges. The omentum was then sutured across the anterior abdominal wall, a glass tube was left in the pouch of Douglas, and the parietal wound was closed. The abdomen was then firmly strapped so as to secure contact as far as possible between the opposed surfaces, and care was taken by frequently pumping at the tube to prevent any such re-accumulation of fluid as might separate the surfaces again. The result in one case was a brilliant success. In the other, the liver was found to be enlarged, smooth on the surface, and not harder than normal; in fact, it was not a case of cirrhosis. Dropsy in this case returned, and the patient died. There was no post-mortem examination. Of the two further cases now reported, the first was operated on in January, 1897, and in January, 1899, he was so well that he had been accepted by the medical adviser of an insurance company as a first-class life. In the other, in addition to a cirrhotic liver, an ovarian tumour was found, and after death the kidneys were seen to be markedly granular. Thus, of the four cases operated on, the two which were uncomplicated cases of hepatic cirrhosis were both cured. The conclusion, then, is arrived at that ascites due to liver cirrhosis can be cured by establishing an efficient anastomotic circulation, and that it is no longer advisable to treat such ascites by repeated tappings if the patient is otherwise sound and in good condition. After one or two tappings have failed, operation offers the best chance of prolonged and useful life.

[1] Lancet, May 27.

